# Nanoparticle Probes for Structural and Functional Photoacoustic Molecular Tomography

**DOI:** 10.1155/2015/757101

**Published:** 2015-11-01

**Authors:** Haobin Chen, Zhen Yuan, Changfeng Wu

**Affiliations:** ^1^State Key Laboratory on Integrated Optoelectronics, College of Electronic Science and Engineering, Jilin University, Changchun, Jilin 130012, China; ^2^Bioimaging Core, Faculty of Health Sciences, University of Macau, Taipa, Macau

## Abstract

Nowadays, nanoparticle probes have received extensive attention largely due to its potential biomedical applications in structural, functional, and molecular imaging. In addition, photoacoustic tomography (PAT), a method based on the photoacoustic effect, is widely recognized as a robust modality to evaluate the structure and function of biological tissues with high optical contrast and high acoustic resolution. The combination of PAT with nanoparticle probes holds promises for detecting and imaging diseased tissues or monitoring their treatments with high sensitivity. This review will introduce the recent advances in the emerging field of nanoparticle probes and their preclinical applications in PAT, as well as relevant perspectives on future development.

## 1. Introduction

PAT, as an emerging powerful modality, has the capability to image the structural and functional information of biological tissues with high resolution and satisfactory imaging depth [1–4]. In PAT, a nonionizing short-pulsed laser source is generally used to illuminate the biological tissues. After the irradiated tissues absorb the light energy, the acoustic waves are produced due to thermoelastic expansion. The acoustic pressure distributions along the tissue surfaces are detected by ultrasound transducers, which can be utilized to generate the functional or structural photoacoustic images. PAT is able to image the optical properties, physiological parameters including deoxyhemoglobin and oxyhemoglobin concentrations, and mechanical parameters such as acoustic velocity of biological tissues. Interestingly photoacoustic molecular imaging can be used to detect biomarkers and reveal specific tumor cells or gene expression [5–8].

PAT based on intrinsic contrast is dominated by the endogenous agents of biological tissues such as hemoglobin, melanin, and water [9]. As a result, the imaging accuracy of PAT for diseased tissues is governed by these contrasts. For example, the diseased tissues scatter or absorb lights differently from those of healthy ones and another noticeable distinction between the two lies in respective background noise. However, the intrinsic optical contrast is probably not sufficient and in many cases may not be disease-specific. As such, it is essential to use the exogenous contrast agents which have some kinds of affinity for the diseased areas via a chemical interaction within the tissues, which is able to provide sensitive and disease-specific detection or monitoring. The use of photoacoustic exogenous agents has the capability of significantly improving the imaging sensitivity, which will greatly extend PAT's applications to areas such as deep-tissue imaging, cell-specific contrast, and molecular imaging [10–13].

In recent years nanoparticle probes have tremendously impacted scientific researches in biomedical imaging, biological diagnostics, and disease treatments [14]. In comparison with other contrast agents, nanoparticles have their very unique merits: (1) the functional properties are able to be tailored via varying their compositions, structures, sizes, and shapes; (2) the huge specific surface area as well as the quantum confinement effect in some systems bestows crucial properties for the preclinical animal study; and (3) the surface of nanoparticle probes is able to be modified to have different functions for multimodality molecular imaging.

To date, different types of nanoparticle probes, including metal (typically Au or Ag) nanoparticles, carbon nanotubes, and superparamagnetic iron oxide nanoparticles as well as semiconductor quantum dots, have been synthesized and adopted for different biomedical applications [15–24]. PAT combined with nanoparticle contrast agents can provide unique structural and functional information at unprecedented levels. For the mini review, the recent advances in the development of nanoparticle probes in PAT are first summarized. To date, inorganic nanoparticles, such as gold nanostructures, carbon nanotubes, iron oxide, semiconductor quantum dots, and upconversion nanoparticles [20], are successfully applied in PAT as contrast agents. In addition, this work also introduces the recent progress in organic nanoparticles such as semiconducting polymer dots that show improved performance in structural and functional photoacoustic molecular tomography.

## 2. Inorganic Nanoparticle Probes for PAT

### 2.1. Metal Nanostructures

Metal nanoparticles have been employed for photoacoustic molecular tomography because of the biocompatibility, easy modification for targeting, minimized toxicity, and localized surface plasmon resonance peak as well as enhanced optical signals in near-infrared spectral regions [25–27]. In addition, while the intrinsic fluorescence signals from metal nanoparticles are not very strong, certain types of metal clusters and nanoparticles can be employed for fluorescent imaging or PAT. So far six types of metal nanostructures have been utilized as nanoparticle probes for PAT for in vivo tests, including nanospheres [28, 29], nanorods [30, 31], nanocages [32], nanoshells [33], nanobeacons [34], and nanoplates [35]. The basic preparation and in vivo and in vitro investigations for gold nanostructures in PAT have been described in detail by previous work [3]. Here we only focus on its recent advances in biomedical fields. It is widely recognized that the determination of tumor margin during the surgical resection is essential for the prevention of tumor recurrences [36, 37]. Kircher et al. have used gold nanospheres for both PAT and surface-enhanced Raman spectroscopy (SERS). Their results revealed that multimodal molecular imaging (MRI-PAT-SERS) using the multifunctional nanoprobes can help quantify the tumor margins of living mice with high accuracy [38]. In addition, gold nanorods (GNRs) have several advantages that motivate their biomedical applications for PAT [39–41]. For example, GNRs have enhanced optical absorption cross section, which is able to generate strong photoacoustic signals and produce minimized uptakes within the reticuloendothelial systems. Accordingly, molecular imaging agents using the nanorods as a passively targeted probe were proposed, in which the probes allowed for presurgical tumor imaging for locoregional staging by PAT and intraoperative imaging of tumor margins to remove the tumors completely by SERS [31].

Nanoshells, particularly gold nanoshells, have been demonstrated as valuable vehicles for ex vivo and preclinical studies. Nanoshells coated with polyethylene glycol (PEG) can improve the biocompatibility and the circulating lifetime though the animal body. Nanoshells have been used as an intravascular contrast agent for optical coherence tomography (OCT) and PAT to identify the vascular structures of the brains [1]. Nanoshells can be conjugated to certain biomolecules that allow for specific targeting to malignant tumors because the protocol utilized to conjugate biomolecules to the surface of the gold has been well established. Nanoshells can also provide the early detection of diseases and can be used as tools for the treatment of disorders or diseases, which includes the use of hollow structures as carriers of different antitumor drugs, the development of scattering nanoshells as nanoprobes for OCT, and the implementation of absorbing nanoshells in near-infrared thermal therapy of tumors [42].

### 2.2. Carbon Nanotubes

**Figure 1:**
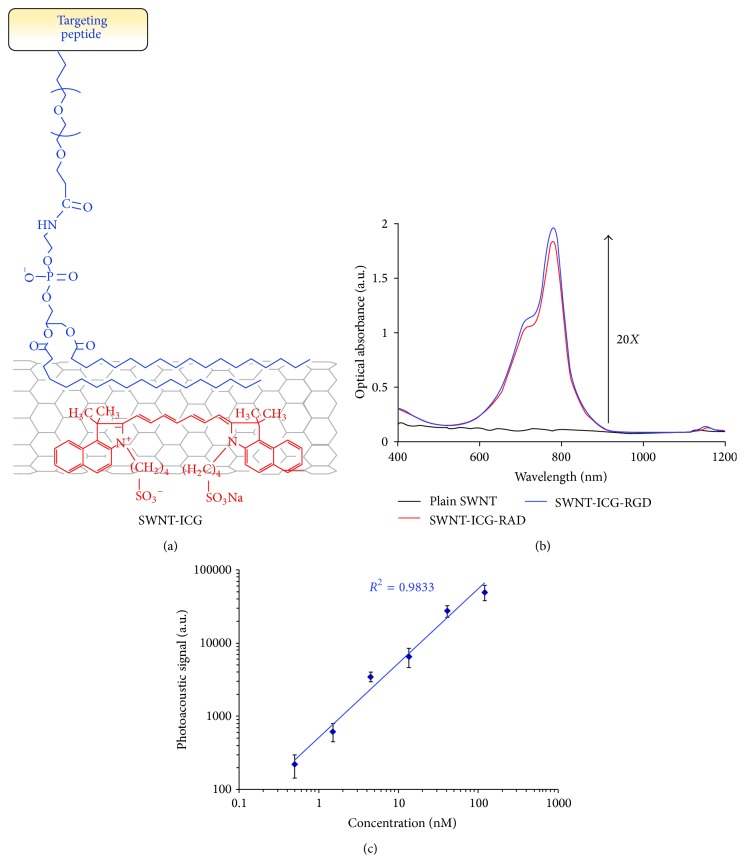
ICG dye-enhanced SWNT probes for photoacoustic imaging. (a) A schematic of a SWNT-ICG particle with ICG molecules (red) attached to the SWNT surface via noncovalent *π-π* stacking bonds. The targeting peptide was attached to SWNT via a PEGylated phospholipid. (b) The absorption spectra from different probes including SWNT-ICG-RAD (red), plain SWNT (black), and SWNT-ICG-RGD (blue). The absorption intensity of ICG dye-SWNTs particles was much higher (over 20 times) than that of plain SWNT at 780 nm. The absorption spectra between SWNT-ICG-RAD and SWNT-ICG-RGD were very similar, which validated that the peptide conjugation should not significantly perturb the photoacoustic signal. (c) The photoacoustic signals of SWNT-ICG was proportional to the probe concentrations (*R* ^2^ = 0.9833). Reproduced with permission from [21].

Single-walled carbon nanotubes have been extensively investigated and used as nanoparticle probes for photoacoustic molecular tomography [17, 43–45]. The broad absorption spectrum of single-walled carbon nanotubes (SWCNTs) can cover the optical window of biological tissue, therefore yielding a strong photoacoustic signal. de La Zerda et al. demonstrated that SWCNTs conjugated with cyclic Arg-Gly-Asp peptides can be adopted as nanoparticle probes for photoacoustic molecular imaging of cancers [17]. The photoacoustic signals from the targeted nanotubes in mice bearing tumors were found to be eight times higher than that from the nontargeted ones. In addition, SWCNTs can be further modified to improve their performance for PAT. For instance, Indocyanine Green dye enhanced SWCNTs (SWNT-ICG) were proposed to generate photoacoustic signals that can provide a remarkably high optical contrast for in vivo animal study [21]. In particular, the ultrahigh surface area of the nanotubes has highly efficient loading of aromatic molecules such as ICG on the nanotube surface, which can create a new sort of photoacoustic probes including SWNT-ICG-RGD (Figure 1(a)). The optical absorption spectrum for the novel SWNT-ICG nanoparticles shows that the SWNT-ICG particles can perform a 20-fold higher absorbance at its peak absorbance (780 nm) when compared to plain SWCNTs (Figure 1(b)). They also built a nonabsorbing and nonscattering agarose phantom with inclusions of SWNT-ICG-RGD with increased concentrations from 0.5 nM to 121.5 nM. They found that the photoacoustic signals generated by the SWNT-ICG-RGD particles correlated well with the nanoparticle concentrations (Figure 1(c)). Finally, they also revealed that the new nanoparticle probes could detect cancer cells 20 times fewer than those by previously reported SWCNTs [21].

### 2.3. Superparamagnetic Iron Oxide Nanoparticles

Superparamagnetic iron oxide nanoparticles (SPIONs) are FDA approved nanoprobes for MRI, which can also be used for nanoparticles probes for photoacoustic molecular tomography due to their perfect biosafety profiles [18, 46–49]. For example, recent work revealed that the contrast agents composed of SPIONs cores coated with silica showed their potentials as nanoparticle probes for PAT when irradiated with 1064 nm laser sources [46]. For this work, they employed a modulated continuous wave laser to access the maximum depth characterization of silica-coated SPION and they also tested the optical stability of the nanoprobes within different solvents in PAT. Importantly they found that the minimum detectable concentration of the silica-coated SPION at depths of 5 mm and 10 mm inside the intralipid was ~0.17 and ~0.23 mg/mL, respectively [46].

### 2.4. Quantum Dots

Quantum dots (Qdots) generally refer to semiconductor nanocrystals when the physical dimensions are much smaller than the exciton Bohr radius [50, 51]. Unlike bulk counterparts, these tiny structures exhibit broad excitation spectra which range from ultraviolet to near-infrared regions and discrete energy levels that are determined primarily by their sizes and chemical compositions [52, 53]. When compared to organic dyes or fluorescent proteins, Qdots exhibit several important properties including high absorption coefficients and enhanced photostability [54]. Due to the wide absorption bands and narrow emission bands, Qdots are considered as extraordinary contrast agents for fluorescence imaging and multiplexed detections [55]. Importantly, the large surface area of Qdots enables the design of multifunctional probes for multimodal molecular imaging. Qdots are playing an essential role as versatile labels for biomedical imaging. Interestingly, Qdots are also used as nanoparticle probes for PAT [19, 56, 57]. For example, Shashkov et al. demonstrated the applications of Qdots in PAT and photothermal microscopy [19]. By using a nanosecond pulsed laser, the bubble formation phenomena were performed using an advanced multifunctional microscope that integrated fluorescence and photoacoustic and photothermal imaging. They found that Qdots can be used as multifunctional contrast agents and sensitizers for multimodal molecular imaging and photothermal therapy [19].

**Figure 2:**
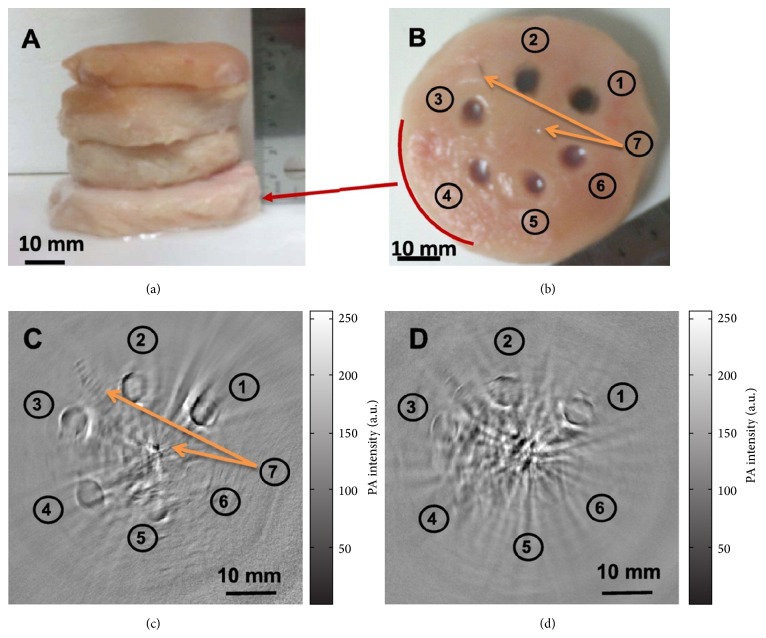
Photoacoustic molecular tomography of deep-embedded targets based on CuS Qdot probes. The agarose gels that contained CuS Qdots were placed in the background of chicken breast muscle with different chicken breasts stacked. (a) showed the configuration of chicken breast muscle stacked. (b) Photograph of chicken breast muscle that had the targets with CuS Qdots of (1) 100 *μ*g/mL (2 OD), (2) 50 *μ*g/mL (1 OD), (3) 25 *μ*g/mL (0.5 OD), (4) 12.5 *μ*g/mL (0.25 OD), (5) 6.25 *μ*g/mL (0.125 OD), (6) gel without contrast agent, and (7) two needle tips at the center and 11 o'clock position. The photoacoustic image was shown at the bottom low with a depth of (c) 25 mm and (d) 50 mm. Reproduced with permission from [22].

Semiconductor copper sulfide (CuS) has received extensive attention for its applications in catalysis and photovoltaics. Recently different gold nanostructures have been developed for the combination applications of PAT and chemotherapy [58, 59]. However, the near-infrared absorbance peaks of gold nanostructures, such as GNRs, would disappear after 1 h laser irradiation at a low power density [60]. Interestingly, the near-infrared absorption of CuS Qdots, derived from the d-d transition of Cu^2+^ ions, seldom changes with their morphologies [61]. A few modalities have been implemented for the development of CuS Qdots [62, 63]. For example, Zhou et al. conducted thioglycolic acid-stabilized CuS Qdots and showed their application for photothermal destruction of tumor cells in vitro using a near-infrared laser beam [64]. They also found that the absorption peak could be tuned toward longer wavelengths by simply adjusting the stoichiometric ratio between CuCl_2_ and Na_2_S [22]. In their study, the average diameter of the CuS Qdots was 11 nm and the molar absorption coefficient at 1064 nm was estimated to be 2.6 × 10^7^ cm^−1^ M^−1^. The strongest absorption peak at 1064 nm implies that CuS Qdots are encouraging entrant for PAT contrast enhancement. Employing a Nd:YAG laser at a wavelength of 1064 nm for PA excitation, PAT clearly visualized CuS Qdots in mouse brain and rat lymph nodes. Furthermore, agarose gel containing CuS Qdots embedded in chicken breast at a depth of ~5 cm could be promptly imaged with an in-plane imaging resolution of ~800 *μ*m and a sensitivity of ~0.7 nmol per imaging voxel (Figure 2). Their work showed that it is possible to image lesions in the human breast at a depth of up to 40 mm with imaging resolution and sensitivity similar to that attained with CuS Qdots in chicken breast muscles. Besides the breast, lesions located in other anatomic sites such as the skin, arm or leg, head and neck, and lymph nodes may also be detected with the next generation of PAT devices equipped with more powerful 1064 nm lasers and a more sensitive ultrasonic detection array.

### 2.5. Upconversion Nanoparticles

**Figure 3:**
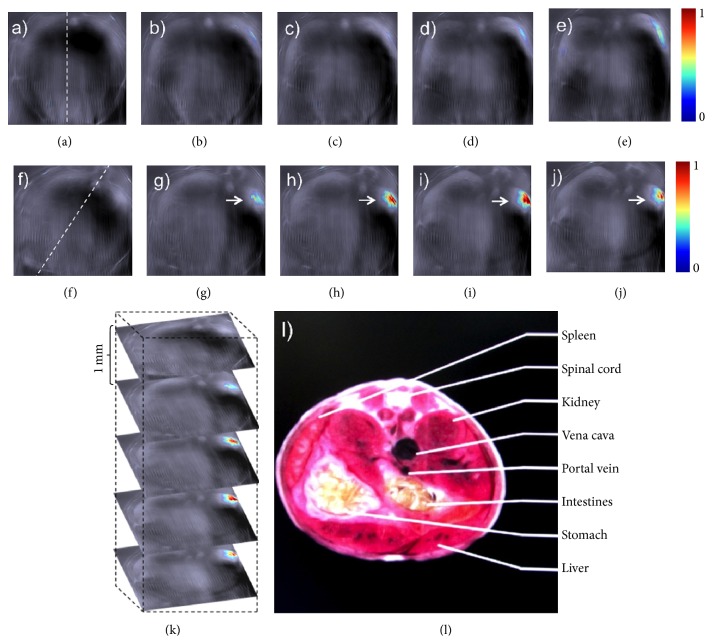
Photoacoustic molecular imaging of mice in vivo based on the lanthanide upconversion nanoparticles (UC-*α*-CD). ((a)–(e)) Generated slice images of live mice before UC-*α*-CD injection. ((f)–(j)) 35 minutes after injection. Dashed lines in (a) and (f) displayed positions of the mouse while (g)–(j) showed the localized UC-*α*-CD. (k) 3D rendering of scanned region. (l) Schematic sections related to analysis region. Reproduced with permission from [20].

Recently upconversion nanoparticles (UCNPs) such as NaYF_4_ codoped with lanthanide ions have been developed as potential nanoparticle probes for biomedical imaging [65, 66]. Maji et al. found that the NaYF_4_:Yb^3+^, Er^3+^ UCNPs with *α*-cyclodextrin (UC-*α*-CD) in aqueous conditions were able to exhibit luminescence quenching when excited at 980 nm. The nonradiative relaxation can result in an unprecedented and high photoacoustic signal. In vivo localization of UC-*α*-CD was conducted using PAT in live mice (Figure 3) [20]. The luminescence quenching of UC-*α*-CD in aqueous solution due to nonradiative relaxation of the excited states will generate intrinsic heat generation during the upconversion process under 980 nm excitation. UC-*α*-CD has been identified to be noncytotoxic and suitable for in vivo PAT as shown by cytotoxicity studies. Figure 3 presented the photoacoustic images generated in real time before and 35 min after intravenous injection of UC-*α*-CD in the thoracic region of the anesthetized mouse. Contrast enhancement was observed for the images before and after injection. It was also found from the reconstructed images in Figure 3 that, compared to other contrast agents, the developed UC-*α*-CD material has the advantages of easy preparation, sharp emission bandwidth, long lifetime, high photostability, low biotoxicity, and more importantly less background autofluorescence [67–70], which makes it an excellent nanoparticle probe for PAT.

## 3. Organic Nanoparticle Probes for PAT

### 3.1. Indocyanine Green-Loaded Photoacoustic Nanodroplets

**Figure 4:**
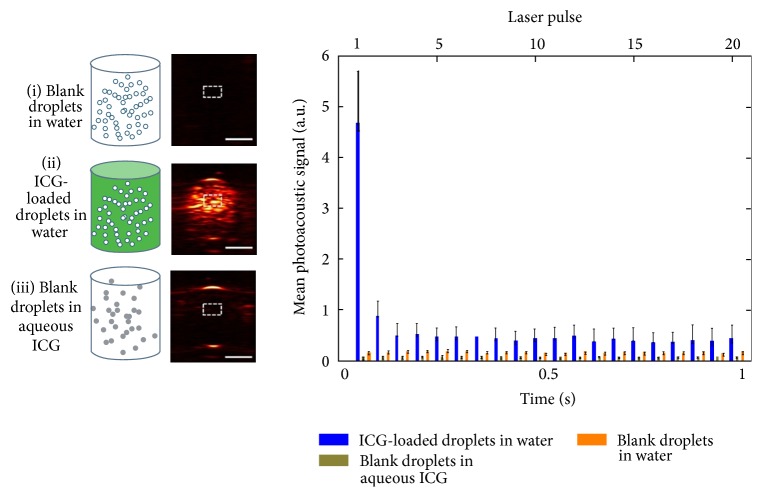
Photoacoustic molecular imaging of ICG-loaded droplets. (i) Blank droplets in water, (ii) ICG-loaded droplets in water, and (iii) blank droplets in aqueous ICG. The right panel plotted the mean photoacoustic intensity identified in the defined ROI. Error bar represents the mean ± standard deviation. *N* ≥ 3 for all reported values; scale bar = 2 mm. Reproduced with permission from [23].

Over the last few years, perfluorocarbon (PFC) nanodroplets have been developed into powerful probes for optical molecular imaging as well as image-guided treatments [71, 72]. For instance, high-intensity ultrasound pulses have been used to generate gas microbubbles according to the phase transitions of liquid PFC. Newly, PFC nanodroplets with encapsulated plasmonic nanoparticles were developed as probes for PAT [73]. In terms of accelerated clinical translation, Hannah et al. developed ICG-loaded PFC nanodroplets, which are identified as nontoxic, biocompatible, and safe materials [23]. The contrast enhancement via droplet vaporization was observed for PAT after the initial laser pulse, and the mean signals were determined over several pulses (Figure 4). They also evaluated the quality enhancement of PAT via the analysis of imaging contrast. Upon irradiation, the PA image contrast was 36 (au), and the contrast-to-noise ratio (CNR) was 51 dB when compared to the 1.1 (au) and 19 dB from blank droplets. They also investigated how the increased ambient temperature would affect the change of PAT imaging contrast. They found that, with increased temperatures, the nanodroplets will generate enhanced photoacoustic signals upon vaporization.

### 3.2. Semiconductor Polymer Dots

Semiconducting polymer nanoparticles (Pdots or SPNs) have recently generated tremendous interests as a novel class of contrast agents for biological imaging [74]. They exhibit several important virtues such as their extraordinary fluorescence brightness, fast emission rate, excellent photostability, and nonblinking and nontoxic features [75–81]. In particular, we recently developed several influential approaches for introducing functional groups, controlling the surface chemistry of Pdots, and employing these novel nanoparticle probes for cellular labeling and in vivo imaging [82–84]. These superior properties of Pdots over other fluorescent probes have established their enormous potential in biology and medicine as highly bright in vitro and in vivo probes.

**Figure 5:**
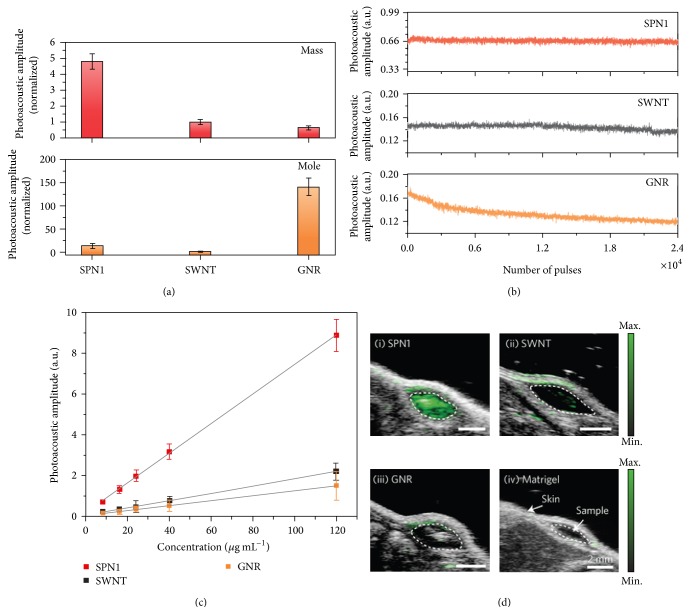
Differences on photoacoustic properties generated from conjugated polymer nanoparticles (SPN1), carbon nanotubes (SWCNTs), and gold nanorods (GNRs). (a) The photoacoustic intensity produced by different nanoparticles based on the same mass (25 *μ*g mL^−1^) (top) and molar (48 nM) (bottom) concentrations in an agar phantom. (b) The photoacoustic intensity regarding indicated nanoparticles in agar phantoms versus the number of laser pulses. (c) The photoacoustic intensity of the nanoparticle-matrigel targets (inclusions) (30 *μ*L) in the subcutaneous dorsal space of living mice as a function of nanoparticle mass concentration. (d) The overlaid photoacoustic/ultrasound images of the nanoparticle-matrigel targets in mice at a concentration of 8 *μ*g mL^−1^. Reproduced with permission from [24].

**Figure 6:**
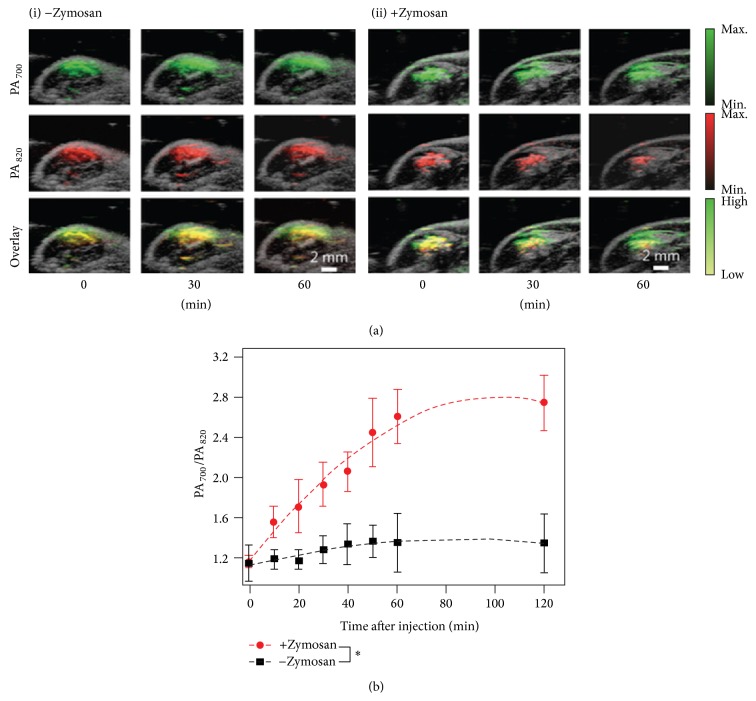
In vivo photoacoustic molecular tomography of reactive oxygen species (ROS) generation in a mouse model of acute oedema by using a ratiometric photoacoustic probe (RSPN). (a) The combined photoacoustic/ultrasound images in the thigh of living mice (*n* = 3) with respect to saline-treated (i) and zymosan-treated (ii) protocols. RSPN was injected into the thigh 20 min after zymosan treatment. (b) The ratio of photoacoustic signals generated between the wavelength of 700 and 820 nm (PA_700_/PA_820_) after RSPN injection. Reproduced with permission from [24].

Pu et al. have developed a new class of near-infrared SPN probes for in vivo photoacoustic molecular imaging [24]. SPN can generate stronger signals than SWCNTs or GNRs on a per mass basis, permitting whole-body lymph node photoacoustic mapping in living mice at a low systemic injection mass. Semiconducting polymers have been originally developed for a wide variety of optoelectronic devices. These purely organic SPNs for PAT have a unique set of advantages that derive from the light-harvesting polymers including the large mass extinction coefficients and excellent photostability. These merits make SPNs superior PAT probes for generating strong and photostable photoacoustic signals in the near-infrared region when compared to SWCNT and GNR. At the same mass concentration, the intrinsic photoacoustic amplitude of the SPN at 700 nm can be over five times higher than that from SWCNT or GNR (Figure 5). Such high photoacoustic brightness in combination with its favorable size enables the efficient PAT of major lymph nodes in living mice with high sensitivity after a single intravenous administration of a small amount of SPNs. With the properties such as the narrow photoacoustic spectral profile, good photostability, and reactive oxygen species (ROS) inert photoacoustic signals, they further developed SPN into an activated NIR ratiometric photoacoustic probe for in vivo imaging of reaction oxygen species (ROS) (Figure 6), a hallmark of many pathological processes such as cancer, cardiomyopathy, stroke, and bacterial infections. The SPN-based photoacoustic probes effectively detect ROS and exhibit great enhancements in ratiometric photoacoustic signals (PA_700_/PA_820_) of 25, 7.3, and 2.7 times in solution, in cells, and in living mice (Figure 6), respectively.

## 4. Conclusions 

In this review, recent advances of both inorganic and organic nanoparticle probes for structural and functional photoacoustic tomography have been highlighted. The use of multifarious nanoprobes for various in vitro, ex vivo, and in vivo tests represents a surging trend in nanobiotechnology and nanomedicine. However, inorganic nanoparticles have the characteristic of being nonbiodegradable, which could accumulate within the animal body for a relatively long time. Importantly, the issue on the long-term toxicity is yet not resolved for preclinical and clinical investigations. In terms of UCNPs, the efficiency of the UC process would be much lower due to the solvent relaxation problem in aqueous conditions, which should significantly affect their potential applications for in vivo photoacoustic molecular tomography. Alternatively, organic nanoparticles including semiconducting polymers show good biocompatibility as demonstrated in different cellular assays [85–87]. In the coming years, we expect a more widespread study and application of these probes with potential clinical translations, in the areas such as deep-tissue imaging, molecular diagnosis of the disease, image-guided delivery, and targeted nanoparticle drug delivery, monitoring disease progression and outcome of therapy. For the further improvement of the performance of photoacoustic probes, we envision that the development of novel light-harvesting nanoparticle species that have well-controlled surface properties as well as targeting capability and the usage of the probes for combined detection and treatment of diseases will be the most particular areas of interest in the near future.
